# Two Decades of Liver Transplants for Primary Biliary Cholangitis: A Comparative Study of Living Donors vs. Deceased Donor Liver Transplantations

**DOI:** 10.3390/jcm12206536

**Published:** 2023-10-15

**Authors:** Esli Medina-Morales, Mohamed Ismail, Romelia Barba Bernal, Yazan Abboud, Leandro Sierra, Ana Marenco-Flores, Daniela Goyes, Behnam Saberi, Vilas Patwardhan, Alan Bonder

**Affiliations:** 1 Department of Medicine, Rutgers New Jersey Medical School, Newark, NJ 07103, USA; jm2831@njms.rutgers.edu (E.M.-M.); mi345@njms.rutgers.edu (M.I.); ya296@njms.rutgers.edu (Y.A.); 2 Division of Gastroenterology, Hepatology and Nutrition, Beth Israel Deaconess Medical Center, Boston, MA 02215, USA; romelia.barba@ttuhsc.edu (R.B.B.); lsierrac@bidmc.harvard.edu (L.S.); amarenco@bidmc.harvard.edu (A.M.-F.); bsaberi@bidmc.harvard.edu (B.S.);; 3 Department of Medicine, Texas Tech University Health Sciences Center, Lubbock, TX 79430, USA; 4 Section of Digestive Diseases, Yale School of Medicine, New Haven, CT 06510, USA; daniela.goyes@yale.edu

**Keywords:** primary biliary cholangitis, liver transplant, living donor liver transplantation, autoimmune liver disease

## Abstract

Primary biliary cholangitis (PBC) prompts liver transplantation (LT) due to cholestasis, cirrhosis, and liver failure. Despite lower MELD scores, recent studies highlight higher PBC waitlist mortality, intensifying the need for alternative transplantation strategies. Living donor liver transplant (LDLT) has emerged as a solution to the organ shortage. This study compares LDLT and deceased donor liver transplant (DDLT) outcomes in PBC patients via retrospective analysis of the UNOS database (2002–2021). Patient survival, graft failure, and predictors were evaluated through Kaplan–Meier and Cox-proportional analyses. Among 3482 DDLTs and 468 LDLTs, LDLT showed superior patient survival (92.3%, 89.1%, 87.6%, 85.0%, 77.2% vs. 91.5%, 88.3%, 86.3%, 82.2%, 71.0%; respectively; *p* = 0.02) with no significant graft survival difference at 1-, 2-, 3-, 5-, and 10-years post-LT (91.0%, 88.0%, 85.7%, 83.0%, 75.4% vs. 90.5%, 87.4%, 85.3%, 81.3%, 70.0%; respectively; *p* = 0.06). Compared to DCD, LDLT showed superior patient and graft survival (*p* < 0.05). Younger male PBC recipients with a high BMI, diabetes, and dialysis history were associated with mortality and graft failure (*p* < 0.05). Our study showed that LDLT had superior patient survival to DDLT. Predictors of poor post-LT outcomes require further validation studies.

## 1. Introduction

Primary biliary cholangitis (PBC) is characterized by immune-mediated damage to the intralobular biliary tract, culminating in cholestasis, cirrhosis, and eventual liver failure, necessitating liver transplantation (LT) [1]. PBC stands as the foremost autoimmune liver disease, with its incidence and prevalence exhibiting an upward trend over the past two decades [2,3]. While predominantly affecting middle-aged or older women, PBC’s potential underdiagnosis in men and presentation at a later stage have been documented [4,5]. Notably, although pruritus, fatigue, and abdominal discomfort afflict PBC patients, more than 50% remain asymptomatic at initial diagnosis [6].

Historically, in the 1980s, PBC was a prominent indication for LT [7]. Yet, advancements in therapeutic agents have led to a diminished need for LT, as these interventions have attenuated disease progression [8]. Ursodeoxycholic acid (UDCA) constitutes the primary treatment, demonstrating improved transplant-free survival [9]. However, around a third of patients fail to respond adequately to UDCA, a subgroup termed biochemical non-responders. These individuals face a heightened risk of cirrhosis-associated hepatic complications, with a cumulative 10-year incidence of 9.1% [10], increasing their chance of advancing to end-stage liver disease, mandating LT. 

Current LT criteria for PBC rely on a Model for End-Stage Liver Disease–sodium score ≥ 15, total bilirubin ≥ 6 mg/dL, or Mayo risk score ≥ 7.8 [11,12]. An additional, albeit rare, indication includes refractory pruritus unresponsive to medical interventions [13]. PBC patients have been shown to have higher waitlist mortality compared to other liver diseases. For example, in comparison to primary sclerosing cholangitis (PSC), PBC exhibits higher waitlist mortality at the 3-month mark [14]. Furthermore, a study contrasting post-LT outcomes in acute-on-chronic liver failure patients highlights PBC’s heightened 1-year waitlist mortality in comparison to other chronic liver diseases [15]. This discrepancy largely stems from the fact that PBC patients tend to have low MELD scores and from the glaring incongruity between the paucity of available deceased donors and the substantial number of patients awaiting transplantation [7]. This situation underscores the urgency to explore alternative transplantation strategies.

Living donor liver transplant (LDLT) has emerged as a prospective solution to bridge the organ supply–demand gap. Comparing LDLT and deceased donor liver transplant (DDLT) outcomes in PBC patients constitutes a pivotal domain of transplantation research. While DDLT has traditionally prevailed as the standard, LDLT presents numerous advantages, encompassing shortened waiting times, reduced risk of graft dysfunction attributed to diminished cold ischemia time, and the potential for superior graft quality due to meticulous donor selection [16,17]. However, there is a scarcity of data surrounding the safety and long-term results of LDLT, particularly in the context of PBC. Moreover, within the DDLT domain, two distinct subcategories emerge that have expanded the donor pool: donation after circulatory death (DCD) and donation after brain death (DBD) [18]. The comparative outcomes of LDLT in relation to these DDLT subgroups remain relatively underexplored [19]. Discerning the nuanced variations in outcomes across these transplantation modalities within the PBC context is pivotal for well-informed clinical decision-making. Therefore, this study aims to compare LDLT against DDLT (as a group or stratified as DCD and DBD subgroups), elucidating survival outcomes and identifying recipient and donor variables linked to patient mortality and graft failure.

## 2. Materials and Methods

### 2.1. Study Population

A retrospective cohort analysis was conducted on adult patients with PBC who were enlisted in the UNOS Organ Procurement and Transplantation Network (OPTN) database and who subsequently underwent primary LT during the interval spanning from January 2002 to December 2021. We excluded the following patients: those younger than 18 years old, those with prior organ transplantation, a history of multi-organ transplantation, and split/reduced LT. The UNOS, which served as the contractor of the OPTN, supplied the data. Any interpretation and reporting of the data shown in the present study are the responsibility of the author(s) and in no way should be seen as an official policy of or interpretation by the OPTN or the US government. Beth Israel Deaconess Medical Center and Rutgers New Jersey Medical School deemed the UNOS database as de-identified and publicly available, hence not requiring institutional review board approval.

### 2.2. Study Variables

We included variables at the time of candidate listing for LT. Both recipients and donor variables were included and shown in two groups, one LDLT and one DDLT. We included the following recipients’ characteristics: age in years, gender, race, blood type group, body mass index (BMI), the presence of diabetes, the use of mechanical assistance, the use of dialysis the week before LT, portal vein thrombosis (PVT), a history of prior abdominal surgery, MELD score and albumin levels at the time of LT, time on the waiting list, if given MELD exceptions points for hepatocellular carcinoma (HCC), patient location (local, regional, and national), and time of transplant on periods (2002–2010, 2011–2019, and 2020–2021). Donor variables were analyzed, including graft type (LDLT and DDLT stratified as either as donation after circulatory death, DCD, or donation after brain death, DBD), age in years, gender, race, BMI, the presence of diabetes, cause of death (COD) in case of both DCD and DBD (anoxia, trauma, cerebrovascular accident, or other), cold ischemia time (CIT) in hours, sharing region (local, regional, and national), and donor and recipient body surface area (BSA) matching (described as appropriate, too small, or too large). BSA was calculated by the formula described by Mosteller [20].

### 2.3. Outcomes Definitions

The primary aim of the present study was to analyze the patient’s and graft’s survival after liver transplantation. Patient survival was defined as the length of time from the date of LT until the date of death or last follow-up. Graft survival was defined as the length of time from the date of LT until the date of graft failure, last follow-up, or the need for a re-LT.

### 2.4. Statistical Analysis

We summarized categorical variables using frequencies and percentages and conducted a chi-square test as a comparative measure. If categorical variables had missing data, our analysis did not include them. Continuous variables were summarized as means and standard deviations or medians and interquartile ranges (IQRs), utilizing ANOVA or the Kruskal–Wallis test based on the distribution of the variable. The median or mean value was assigned in case of missing values in continuous variables.

Survival outcomes were compared utilizing the Kaplan–Meier method. Graft type was compared between LDLT and DDLT. The latter was further stratified into DBD and DCD, and an additional comparison was conducted among LDLT, DBD, and DCD. The log-rank test was employed to assess survival differences across these groups. Stepwise Cox proportional hazard regression models were employed to identify predictors influencing patient and graft survival, adjusted for recipient and donor characteristics. Variables that exhibited statistical significance (recipient age, male gender, BMI, diabetes mellitus, recent use of dialysis, PVT, period of transplant, donor age, national region, and other donor race) or clinical relevance (such as age at transplantation, gender, race, diabetes mellitus, and MELD score) were incorporated into the final model. Statistical significance was defined using a bivariate level (partial regression of 0.1 and partial elimination of 0.05). The statistical analysis was executed using Stata version 17.0 MP (StataCorp LP, College Station, TX, USA).

## 3. Results

### 3.1. Recipients and Donor Characteristics

Over a span of 20 years, a total of 3950 LTs were conducted, comprising 3482 DDLTs (3251 DBD and 231 DCD) and 468 LDLTs. When contrasted with recipients of DDLTs, those undergoing LDLTs exhibited significant differences. LDLT recipients were notably younger (*p* < 0.001), predominantly White (*p* < 0.001), and had the blood type O positive (*p* = 0.004). Additionally, they demonstrated higher levels of serum albumin (*p* = 0.014) and a longer duration on the waiting list (*p* < 0.001). In terms of their characteristics, LDLT recipients had lower body mass indices (BMIs) (*p* < 0.001) and fewer MELD exception score points attributed to hepatocellular carcinoma (HCC) (*p* < 0.001). Moreover, they exhibited a lower likelihood of having undergone dialysis in the week prior to transplantation (*p* < 0.001) or previous abdominal surgeries (*p* = 0.007). For more comprehensive insights into the recipients and donor attributes, please refer to Table 1.

### 3.2. Graft and Patient Survival

#### 3.2.1. Patients Undergoing LDLTs Showed Superior Patient Survival Compared to DDLT Recipients

**Figure 1:**
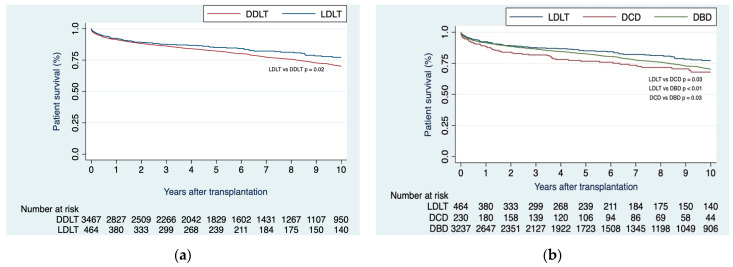
(**a**) Kaplan–Meier curves of patient survival when comparing LDLT vs. DDLT; (**b**) Kaplan–Meier curves of patient survival when comparing LDLT vs. DDLT, with the latter stratified as DBD or DCD.

Upon unadjusted analysis, recipients of LDLTs demonstrated superior survival rates in comparison to those who received DDLTs at 1-, 2-, 3-, 5-, and 10 years (92.3%, 89.1%, 87.6%, 85.0%, 77.2% vs. 91.5%, 88.3%, 86.3%, 82.2%, 71.0%; respectively; *p* = 0.02) (Figure 1a). Patient survival at the same intervals, specifically 1-, 2-, 3-, 5-, and 10 years, proved superior within the LDLT group as contrasted with both the DCD subgroup (92.3%, 89.1%, 87.6%, 85.0%, 77.2% vs. 88.5%, 83.9%, 81.7%, 76.7%, 68.0%; respectively; *p* = 0.03) and DBD subgroup (92.3%, 89.1%, 87.6%, 85.0%, 77.2% vs. 91.0%, 88.6%, 86.5%, 82.6%, 70.3%; respectively; *p* < 0.01). Notably, the DBD subgroup exhibited superior patient survival when compared to the DCD subgroup (91.0%, 88.6%, 86.5%, 82.6%, 70.3% vs. 88.5%, 83.9%, 81.7%, 76.7%, 68.0%; respectively; *p* = 0.03) (Figure 1b).

#### 3.2.2. LDLT Patients Showed Similar Graft Failure Rates Compared to DDLT Recipients

**Figure 2:**
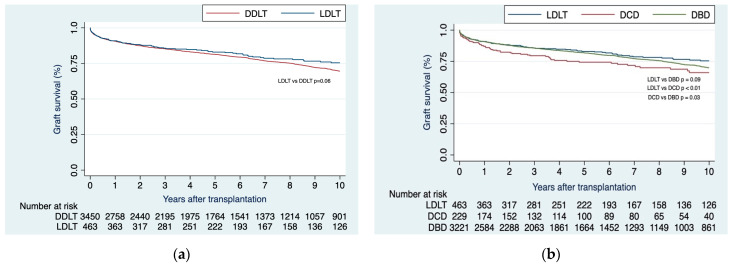
(**a**) Kaplan–Meier curves of graft survival when comparing LDLT vs. DDLT; (**b**) Kaplan–Meier curves of graft survival when comparing LDLT vs. DDLT, with the later stratified as DBD or DCD.

On unadjusted analysis, no distinction emerged in terms of graft survival between recipients of LDLTs and those who underwent DDLTs at 1-, 2-, 3-, 5-, and 10 years (91.0%, 88.0%, 85.7%, 83.0%, 75.4% vs. 90.5%, 87.4%, 85.3%, 81.3%, 70.0%; respectively; *p* = 0.06) (Figure 2a). Correspondingly, graft survival at 1-, 2-, 3-, 5-, and 10-years was similar within the LDLT cohort when compared to the DBD subgroup (91.0%, 88.0%, 85.7%, 83.0%, 75.4% vs. 90.8%, 87.7%, 85.6%, 81.8%, 70.0%; respectively; *p* = 0.09). In contrast, LDLT cohort demonstrated superior graft survival relative to the DCD subgroup (91.0%, 88.0%, 85.7%, 83.0%, 75.4% vs. 87.1%, 82.3%, 79.5%, 74.3%, 66.0%; respectively; *p* < 0.01).

Upon comparing DBD with DCD, the first showed greater graft survival at 1-, 2-, 3-, 5-, and 10 years (90.8%, 87.7%, 85.6%, 81.8%, 70.0% vs. 87.1%, 82.3%, 79.5%, 74.3%, 66%; respectively; *p* = 0.03) (Figure 2b).

### 3.3. Risk Factors of Patient Mortality and Graft Failure

#### 3.3.1. Risk Factors for Patient Mortality

During the multivariable analysis, we identified the following recipient characteristics as risk factors for patient mortality (Table 2): increased age (*p* < 0.001), male gender (*p* = 0.003), the presence of diabetes (*p* = 0.005), higher BMI (*p* = 0.03), the use of dialysis one week before LT (*p* < 0.001), the presence of PVT (*p* = 0.03), and the recipient’s hospital location prior to LT (*p* = 0.004). Moreover, recipient variables linked with improved survival were the transplantation period spanning from 2010 to 2019 (*p* = 0.002) and Region 2 (*p* = 0.001). Similarly, donor factors identified as risk factors for patient survival were both donor age (*p* = 0.007) and other donor race (*p* = 0.03) (Table 2).

#### 3.3.2. Risk Factors for Graft Failure

During the multivariable analysis, we identified the following recipient characteristics as risk factors for graft failure (Table 3): increased age (*p* < 0.001), male sex (*p* = 0.003), the presence of diabetes (*p* = 0.01), the use of dialysis one week before LT (*p* < 0.001), and hospital location of the recipient prior LT (*p* = 0.02). Additionally, recipient variables linked with improved survival were the transplantation period spanning from 2010 to 2019 (*p* < 0.001) and Region 2 (*p* = 0.001). Similarly, advanced donor age was associated with a higher risk of graft failure (*p* < 0.001) (Table 3).

## 4. Discussion

Numerous studies have demonstrated that PBC patients generally exhibit lower MELD scores, consequently diminishing the pool of available deceased donors during their waitlisted period [7]. Despite this, PBC has been shown to experience heightened waitlist mortality in comparison to other liver diseases, sparking debates regarding the prospective requirement of MELD exception points for PBC patients [14,21]. Some centers have contemplated LDLT as a viable option for PBC patients, and multiple studies have indicated survival rates exceeding 80% after 5 years post-LT [19]. Our study found that for unadjusted patient survival, LDLT showed superior outcomes compared to DDLT, whereas no distinction was found in graft survival. When stratifying DDLT as DBD and DCD, we observed that LDLT exhibited superior patient survival outcomes compared to both DBD and DCD, and it outperformed only DCD in terms of graft survival. Notably, DCD exhibited poorer graft and patient survival outcomes.

Our study demonstrated mortality and graft failure rates among PBC patients undergoing LT that align consistently with findings from prior research. For instance, a study encompassing patients with chronic liver disease who underwent LT and utilized a UNOS database spanning from 1994 to 2009 reported survival rates of 85%, 80.5%, 78.1%, and 71.9% at 1-, 3-, 5- and 10-year intervals [22]. In another dataset, the Scientific Registry of Transplant Recipient (SRTR), Sayiner et al., investigated the post-LT outcomes of PBC patients in comparison to patients with hepatitis C [23]. The results of the study indicated patient mortality rates of 10.9%, 15.7%, and 19.8% at 1-, 3- and 5-years, respectively. Our 20-year period study yielded excellent patient survival rates after DDLT with rates of 91.5%, 88.3%, 86.3%, 82.2%, and 71.0% at 1-, 2-, 3-, 5-, and 10-year intervals.

In an unadjusted analysis comparing LDLT to DDLT, we observed that LDLT demonstrated superior patient survival compared to DDLT, while no distinction was evident in graft survival. A previous study, also conducted within the UNOS database, encompassing patients with autoimmune and cholestatic disease from February 2002 to October 2006, demonstrated that among all recipients, the estimated patient survival rates at 1, 3, and 5 years were 95.5%, 93.6%, and 92.5% for LDLT and 90.9%, 86.5%, and 84.9% for DDLT, respectively (*p* = 0.002) [24]. Estimated graft survival rates at 1, 3, and 5 years were 87.9%, 85.4%, and 84.3% for LDLT and 85.9%, 80.3%, and 78.6% for DDLT, respectively (*p* = 0.123). Another study investigating recipients with autoimmune liver diseases from the European Liver Transplant Registry included 1003 LDLT transplants, where 158 were attributed to PBC patients. In comparing these to DDLT (in this study, DBD), no significant difference emerged (*p* = 0.963) [25]. A Japanese study including 444 PBC patients who underwent LDLT reported an excellent long-term overall survival rate of 76.6% at five years, 71.2% at ten years, and 52.6% at 15 years post-transplantation [26]. Our findings highlight the potential of LDLT as an appealing alternative for PBC patients, particularly in light of the existing constraints on organ availability.

Upon stratifying DDLT as DBD and DCD, we found that LDLT exhibited superior patient survival compared to both groups. No difference was observed in graft survival when comparing LDLT to DBD, although LDLT outperformed DCD. It is worth noting that only a limited number of studies have compared DBD and DCD to LDLT in PBC recipients. Ziogas et al. reported superior unadjusted patient survival on LDLT compared to DBD (*p* < 0.001) but not to DCD (*p* = 0.21) [19]. In contrast, in the same study, LDLT showed better graft survival rates compared to both groups. Upon adjusting for multiple recipients and donor variables, the effect of donor type, whether as DDLT or stratified as DBD and DCD, exhibited no significant difference in either patient survival in PBC recipients. However, the same study identified DCD as an independent risk factor for poor graft survival on the multivariate analysis [19].

Our study has revealed that both recipient and donor age serve as risk factors for patient and graft survival in patients with PBC. Comparable results have been documented in prior research regarding recipient age [23,24,26,27] and donor age [19]. Furthermore, we found that male gender showed inferior LT outcomes. PBC tends to manifest in older men with more severe disease [1,4]. These individuals are less likely to respond effectively to UDCA treatment and exhibit a lower likelihood of experiencing symptoms [7]. Consequently, it is plausible that male PBC patients on the transplant waiting list might have more severe disease and advanced age, thereby increasing the risk of suboptimal outcomes post-LT. However, some studies have failed to establish gender as a significant risk factor for LT outcomes [19,23,24].

Furthermore, our analysis revealed that LT conducted within the time frame of 2011 to 2019 exhibited superior patient and graft survival outcomes during the post-transplant period. However, it is essential to acknowledge that these findings are constrained by the potential limitations stemming from the presence of missing or incomplete data spanning the years of 2002 to 2010 (attributed to the recent establishment of LT in various medical centers) and 2020 to 2021 (owing to the global impact of the COVID-19 pandemic).

The strength of our study relies on the utilization of a large sample size, meticulously collected through a rigorous and systematic approach. Additionally, we opted to further stratify DDLT into distinct categories, namely DBD and DCD, a comparison that has only been explored in one other study [19]. However, it is important to note that this comparison study encompassed including another cholestatic liver disease such as PSC, which has a distinct underlying pathogenesis, prognosis, and course of disease after LT [28]. With the implementation of the MELD 3.0, which introduces adjustments to the MELD score, particularly including female sex, we anticipated changes in the waiting list outcomes and post-LT outcomes after DDLT or LDLT in PBC recipients in the subsequent years [29].

Nonetheless, while the utilization of a comprehensive database offers valuable insights, there are inherent limitations. First, there exist underlying baseline recipient and donor characteristics between LDLT and DDLT that might not have been fully captured due to the lack of granular data in the UNOS database. Second, our findings are retrospective in nature. Third, our study lacked the intention to treat and did not consider the dropout rates, which we assumed to be higher in PBC recipients. Fourth, certain pertinent clinical information relevant to PBC outcomes in LT is absent. For instance, details like whether the criteria for listing involved intractable pruritus or severity of liver failure or if patients were placed on second-line medication (such as obeticholic acid) or off-label medications (fibrates) prior to listing remain undisclosed.

## 5. Conclusions

LDLT showed improved patient survival in comparison to DDLT at 1-, 2-, 3-, 5-, and 10-year intervals. When stratifying DDLT into DCD and DBD, LDLT also showed lower mortality rates. LDLT emerges as an appealing choice for patients with PBC, particularly given the propensity to exhibit fewer symptoms and consequently a reduced likelihood of being captured by the MELD score criteria. Regardless of graft type, poorer post-LT outcomes were observed in younger male PBC recipients with a higher BMI and a history of diabetes and recent dialysis. To further validate these findings, more prospective studies incorporating granular data are warranted.

## Figures and Tables

**Table 1:** Recipient and donor characteristics by graft type.

Variable	LDLT (*n* = 464)	DCD (*n* = 230)	DBD (*n* = 3237)	*p*-Value
**RECIPIENT CHARACTERISTICS**				
**Age (years)**	55 (48, 62)	59 (52, 65)	57 (50, 64)	<0.001
**Gender, *n* (%)**				
**Female**	397 (85.6%)	186 (80.9%)	2669 (82.5%)	0.19
**Male**	67 (14.4%)	44 (19.1%)	568 (17.5%)	
**Race, *n* (%)**				
**White**	375 (80.8%)	174 (75.7%)	2319 (71.6%)	<0.001
**Black**	14 (3.0%)	18 (7.8%)	255 (7.9%)	
**Hispanic**	67 (14.4%)	29 (12.6%)	521 (16.1%)	
**Asian**	3 (0.6%)	6 (2.6%)	94 (2.9%)	
**Other**	5 (1.1%)	3 (1.3%)	48 (1.5%)	
**DM, *n* (%)**	51 (11.0%)	36 (15.7%)	478 (14.8%)	0.081
**Blood type, *n* (%)**				
**O**	218 (47.0%)	92 (40.0%)	1498 (46.3%)	0.004
**A**	185 (39.9%)	87 (37.8%)	1165 (36.0%)	
**B**	53 (11.4%)	41 (17.8%)	407 (12.6%)	
**AB**	8 (1.7%)	10 (4.3%)	167 (5.2%)	
**BMI**	25.2 (22.7, 28.9)	26.7 (23.6, 30.7)	26.3 (23.2, 30.3)	<0.001
**Life support, *n* (%)**				
**No**	464 (100.0%)	227 (98.7%)	3198 (98.8%)	0.058
**Yes**	0 (0.0%)	3 (1.3%)	3 (1.3%)	
**Dialysis during the week before LT**	2 (0.4%)	22 (9.6%)	325 (10.0%)	<0.001
**Laboratory MELD score at LT**	15 (11, 19)	23 (17, 30)	22.7 (17, 30)	<0.001
**Albumin Level at LT**	3 (2.6, 3.5)	2.9 (2.5, 3.4)	2.9 (2.5, 3.4)	0.014
**Previous** **abdominal surgery**	250 (53.9%)	149 (64.8%)	1963 (60.6%)	0.007
**Portal vein thrombosis**	39 (8.4%)	31 (13.5%)	357 (11.0%)	0.100
**UNOS/OPTN region where listed/** **transplanted**				
**1**	32 (6.9%)	3 (1.3%)	89 (2.7%)	<0.001
**2**	80 (17.2%)	37 (16.1%)	289 (8.9%)	
**3**	5 (1.1%)	31 (13.5%)	597 (18.4%)	
**4**	23 (5.0%)	8 (3.5%)	338 (10.4%)	
**5**	93 (20.0%)	21 (9.1%)	461 (14.2%)	
**6**	1 (0.2%)	3 (1.3%)	119 (3.7%)	
**7**	83 (17.9%)	29 (12.6%)	293 (9.1%)	
**8**	30 (6.5%)	17 (7.4%)	239 (7.4%)	
**9**	64 (13.8%)	16 (7.0%)	179 (5.5%)	
**10**	28 (6.0%)	41 (17.8%)	361 (11.2%)	
**11**	25 (5.4%)	24 (10.4%)	272 (8.4%)	
**MELD exception points were given for HCC**	6 (1.3%)	18 (7.8%)	297 (9.2%)	<0.001
**Total days on waiting list/including inactive time**	170 (83, 371)	120 (20, 380)	111 (26, 373)	<0.001
**Time periods**				
**<2011**	196 (42.2%)	84 (36.5%)	1446 (44.7%)	0.089
**2010–2019**	205 (44.2%)	110 (47.8%)	1412 (43.6%)	
**≥2020**	63 (13.6%)	36 (15.7%)	379 (11.7%)	
**DONOR CHARACTERISTICS**				
**Age (years)**	36 (28, 45)	39 (27, 53)	45 (28, 58)	<0.001
**Gender, *n* (%)**				
**Female**	256 (55.2%)	104 (45.2%)	1564 (48.3%)	0.011
**Male**	208 (44.8%)	126 (54.8%)	1673 (51.7%)	
**Race, *n* (%)**				
**White**	372 (80.2%)	148 (64.3%)	2137 (66.0%)	<0.001
**Black**	14 (3.0%)	48 (20.9%)	502 (15.5%)	
**Hispanic**	62 (13.4%)	28 (12.2%)	462 (14.3%)	
**Asian**	6 (1.3%)	5 (2.2%)	75 (2.3%)	
**Other**	10 (2.2%)	1 (0.4%)	61 (1.9%)	
**DM, *n* (%)**	0 (0.0%)	31 (13.5%)	383 (11.8%)	<0.001
**BMI**	26.3 (23.8, 28.3)	26.2 (23, 30.4)	26.0 (22.7, 29.7)	0.51
**Cause of death**				
**Anoxia**	0 (0.0%)	125 (54.3%)	820 (25.3%)	<0.001
**CVA**	0 (0.0%)	45 (19.6%)	1254 (38.7%)	
**Head trauma**	0 (0.0%)	55 (23.9%)	1066 (32.9%)	
**Other**	464 (100.0%)	5 (2.2%)	97 (3.0%)	
**Cold ischemia time (hours)**	1.7 (1, 2.7)	6.0 (4.5, 7.5)	6.0 (4.8, 7.7)	<0.001
**Sharing region**				
**Local**	464 (100.0%)	150 (65.2%)	2116 (65.4%)	<0.001
**Regional**	0 (0.0%)	51 (22.2%)	822 (25.4%)	
**National**	0 (0.0%)	29 (12.6%)	299 (9.2%)	
**Donor–recipient** **match per BSA**				
**Too small**	0 (0.0%)	3 (1.3%)	61 (1.9%)	0.022
**Appropriate size**	404 (87.1%)	199 (86.5%)	2834 (87.6%)	
**Too large**	60 (12.9%)	28 (12.2%)	342 (10.6%)	

LDLT, living donor liver transplantation; DBD, donation after brain death; DCD, donation after circulatory death; DM, diabetes mellitus; BMI, body mass index; LT, Liver transplantation; MELD, Model for End-Stage Liver Disease; UNOS, United Network for Organ Sharing; OPTN, Organ Procurement and Transplantation Network; HCC, hepatocellular carcinoma; CVA, cerebrovascular accident; BSA, body surface area.

**Table 2:** Univariate and stepwise multivariate Cox proportional hazard analyses of predictors of post-transplant patient survival in PBC recipients.

	Univariate			Multivariate		
	HR	95% CI	*p*-Value	HR	95% CI	*p*-Value
**RECIPIENT CHARACTERISTICS**						
**Recipient Age**	1.32	1.23–1.42	<0.001	1.28	1.20–1.38	<0.001
**Male Gender**	1.31	1.13–1.52	<0.001	1.26	1.08–1.47	0.003
**Recipient race** (White ref.)						
**Black**	0.94	0.74–1.20	0.63			
**Hispanic**	0.86	0.71–1.04	0.11			
**Asian**	0.78	0.48–1.26	0.31			
**Other**	0.90	0.52–1.56	0.71			
**DM**	1.38	1.17–1.63	<0.001	1.27	1.08–1.50	0.005
**Blood type** (O ref.)						
**A**	1.09	0.95–1.25	0.22			
**B**	1.13	0.93–1.36	0.21			
**AB**	1.22	0.92–1.63	0.17			
**BMI**	1.02	1.00–1.03	0.01	1.01	1.00–1.02	0.03
**Life Support**	1.04	0.54–2.00	0.91			
**Dialysis during the week before LT**	1.46	1.18–1.79	<0.001	1.51	1.23–1.87	<0.001
**Laboratory MELD score at LT**	1.00	0.99–1.00	0.28			
**Albumin level at LT**	1.02	0.94–1.11	0.64			
**Previous abdominal surgery**	1.15	1.01–1.30	0.03			
**Portal vein thrombosis**	1.28	1.04–1.56	0.02	1.25	1.02–1.53	0.03
**Region** (Region 1 ref.)						
**2**	1.08	0.74–1.55	0.70	1.36	1.13–1.63	0.001
**3**	0.79	0.55–1.14	0.21			
**4**	0.88	0.60–1.29	0.51			
**5**	0.67	0.46–0.98	0.04			
**6**	0.58	0.36–0.93	0.02			
**7**	0.78	0.53–1.13	0.19			
**8**	0.63	0.42–0.94	0.02			
**9**	0.84	0.56–1.25	0.38			
**10**	0.83	0.57–1.21	0.34			
**11**	0.83	0.56–1.23	0.35			
**MELD exception points were given for HCC**	1.35	1.10–1.65	0.01			
**Time on waiting list**	1.00	1.00–1.00	0.94			
**Period** (2002–2010 ref.)						
**2011–2019**	0.86	0.74–0.99	0.04	0.84	0.74–0.96	0.01
**2020–2021**	1.11	0.77–1.58	0.58			
**DONOR CHARACTERISTICS**						
**Donor Age**	1.01	1.00–1.01	<0.001	1.00	1.00–1.01	0.007
**Male Gender**	1.11	0.99–1.26	0.09			
**Donor race** (White ref.)						
**Black**	1.21	1.01–1.44	0.04			
**Hispanic**	1.03	0.86–1.25	0.73			
**Asian**	1.25	0.84–1.84	0.27			
**Other**	1.58	1.04–2.40	0.03	1.58	1.05–2.40	0.03
**DM**	1.06	1.00–1.13	0.06			
**BMI**	1.01	1.00–1.02	0.15			
**Living Donor**	0.79	0.64–0.97	0.02			
**Graft type** (LDLT ref.)						
**DBD**	1.63	1.19–2.22	<0.001			
**DCD**	1.25	1.02–1.53	0.03			
**Cause of death** (Anoxia ref.)						
**CVA**	1.12	0.94–1.33	0.20			
**Head trauma**	1.03	0.86–1.23	0.77			
**Other**	0.88	0.71–1.10	0.28			
**Cold ischemia time**	1.02	1.01–1.04	0.001			
**Sharing region** (Local ref.)						
**Regional**	0.96	0.83–1.13	0.65			
**National**	1.58	1.27–1.97	0.001	1.38	1.11–1.73	0.004
**Donor–Recipient match as per BSA** (Appropriate ref.)						
**Too small**	1.35	0.87–2.11	0.18			
**Too large**	0.96	0.78–1.18	0.70			

PBC, primary biliary cholangitis; CI, confidence interval; HR, hazard ratio; DM, diabetes mellitus; BMI, body mass index; LT, liver transplantation; MELD, Model for End-Stage Liver Disease; HCC, hepatocellular carcinoma; DBD, donation after brain death; DCD, donation after circulatory death; CVA, cerebrovascular accident; BSA, body surface area.

**Table 3:** Univariate and stepwise multivariate Cox proportional hazard analyses of predictors of post-transplant graft survival in PBC recipients.

	Univariate			Multivariate		
	HR	95% CI	*p*-Value	HR	95% CI	*p*-Value
**RECIPIENT CHARACTERISTICS**						
**Recipient Age**	1.15	1.07–1.22	<0.001	1.12	1.05–1.93	0.001
**Male Gender**	1.29	1.11–1.49	0.001	1.24	1.08–1.44	0.003
**Recipient race** (White ref.)						
**Black**	0.93	0.74–1.18	0.56			
**Hispanic**	0.81	0.68–0.97	0.03			
**Asian**	0.75	0.48–1.19	0.22			
**Other**	0.92	0.55–1.54	0.76			
**DM**	1.28	1.09–1.50	0.003	1.22	1.04–1.44	0.01
**Blood type** (O ref.)						
**A**	1.08	0.95–1.23	0.22			
**B**	1.18	0.99–1.41	0.07			
**AB**	1.21	0.92–1.59	0.18			
**BMI**	1.01	1.00–1.02	0.03			
**Life support**	1.23	0.68–2.23	0.49			
**Dialysis during the week before LT**	1.36	1.11–1.66	0.003	1.46	1.19–1.78	<0.001
**Laboratory MELD score at LT**	0.99	0.99–1.00	0.11			
**Albumin level at LT**	1.04	0.96–1.13	0.36			
**Previous abdominal surgery**	1.10	0.98–1.24	0.12			
**Portal vein thrombosis**	1.18	0.98–1.44	0.09			
**Region** (Region 1 ref.)						
**2**	1.12	0.79–1.59	0.54	1.35	1.14–1.61	0.001
**3**	0.80	0.56–1.13	0.20			
**4**	0.87	0.61–1.26	0.47			
**5**	0.75	0.52–1.07	0.11			
**6**	0.59	0.37–0.94	0.03			
**7**	0.84	0.59–1.21	0.35			
**8**	0.65	0.44–0.95	0.03			
**9**	0.91	0.62–1.33	0.62			
**10**	0.86	0.60–1.24	0.42			
**11**	0.84	0.58–1.22	0.35			
**MELD exception points were given for HCC**	1.22	1.00–1.50	0.05			
**Time on waiting list**	1.00	1.00–1.00	0.59			
**Period** (2002–2010 ref.)						
**2011–2019**	0.80	0.69–0.92	0.002	0.79	0.68–0.90	0.001
**2020–2021**	0.95	0.68–1.31	0.74			
**Donor characteristics**						
**Donor age**	1.01	1.00–1.01	<0.001	1.01	1.00–1.01	<0.001
**Male gender**	1.07	0.95–1.21	0.24			
**Donor race** (White ref.)						
**Black**	1.18	1.00–1.40	0.05			
**Hispanic**	0.97	0.81–1.16	0.71			
**Asian**	1.10	0.74–1.62	0.64			
**Other**	1.35	0.89–2.05	0.15			
**DM**	1.06	0.99–1.12	0.08			
**BMI**	1.01	1.00–1.02	0.19			
**Living donor**	0.94	0.78–1.13	0.53			
**Graft type** (LDLT ref.)						
**DBD**	1.34	1.00–1.79	0.05			
**DCD**	1.04	0.87–1.26	0.64			
**Cause of death** (Anoxia ref.)						
**CVA**	1.14	0.96–1.35	0.13			
**Head trauma**	1.04	0.88–1.24	0.63			
**Other**	1.04	0.85–1.28	0.70			
**Cold ischemia time**	1.02	1.01–1.04	0.01			
**Sharing region** (Local ref.)						
**Regional**	0.94	0.81–1.09	0.39			
**National**	1.47	1.19–1.82	<0.001	1.28	1.04–1.59	0.02
**Donor–Recipient match as per BSA** (Appropriate ref.)						
**Too small**	1.36	0.89–2.07	0.16			
**Too large**	0.94	0.77–1.15	0.55			

PBC, primary biliary cholangitis; CI, confidence interval; HR, hazard ratio; DM, diabetes mellitus; BMI, body mass index; LT, Liver transplantation; MELD, Model for End-Stage Liver Disease; HCC, hepatocellular carcinoma; DBD, donation after brain death; DCD, donation after circulatory death; CVA, cerebrovascular accident; BSA, body surface area.

## Data Availability

Data can be requested at: https://unos.org/data/ (accessed on 27 August 2023).
